# Gene excavation and expression analysis of CYP and UGT related to the post modifying stage of gypenoside biosynthesis in *Gynostemma pentaphyllum* (Thunb.) Makino by comprehensive analysis of RNA and proteome sequencing

**DOI:** 10.1371/journal.pone.0260027

**Published:** 2021-12-07

**Authors:** Yangmei Zhang, Qicong Chen, Yuanheng Huang, Ruiqiang Zhao, Jian Sun, Xidong Yuan, Huiming Xu, Huiyu Liu, Yaosheng Wu

**Affiliations:** 1 Key Laboratory of Biological Molecular Medicine Research of Guangxi Higher Education, Department of Biochemistry and Molecular Biology, School of Basic Medical Sciences, Guangxi Medical University, Nanning, Guangxi province, China; 2 Department of Nursing, Sichuan Nursing Vocational College, Sichuan province, China; 3 School of Biomedical Science and Engineering, South China University of Technology, Guangzhou, Guangdong province, China; Chinese Academy of Medical Sciences and Peking Union Medical College, CHINA

## Abstract

Previous studies have revealed that gypenosides produced from *Gynostemma pentaphyllum* (Thunb.) Makino are mainly dammarane-type triterpenoid saponins with diverse structures and important biological activities, but the mechanism of diversity for gypenoside biosynthesis is still unclear. In this study, a combination of isobaric tags for relative and absolute quantification (iTRAQ) proteome analysis and RNA sequencing transcriptome analysis was performed to identify the proteins and genes related to gypenoside biosynthesis. A total of 3925 proteins were identified by proteomic sequencing, of which 2537 were quantified. Seventeen cytochrome P450 (CYP) and 11 uridine 5’-diphospho-glucuronosyltransferase (UDP-glucuronosyltransferase, UGT) candidate genes involved in the side chain synthesis and modification of gypenosides were found. Seven putative CYPs (CYP71B19, CYP77A3, CYP86A7, CYP86A8, CYP89A2, CYP90A1, CYP94A1) and five putative UGTs (UGT73B4, UGT76B1, UGT74F2, UGT91C1 and UGT91A1) were selected as candidate structural modifiers of triterpenoid saponins, which were cloned for gene expression analysis. Comprehensive analysis of RNA sequencing and proteome sequencing showed that some CYPs and UGTs were found at both the transcription and translation levels. In this study, an expression analysis of 7 CYPs and 5 UGTs that contributed to gypenoside biosynthesis and distribution in *G*. *pentaphyllum* was performed, providing consistent results that will inspire more future research on vital genes/proteins involved in gypenoside biosynthesis.

## Introduction

*Gynostemma pentaphyllum* (Thunb.) Makino, a slender creeping or climbing plant of the genus *Gynostemma* of the *Cucurbitaceae* family, possesses multiple medicinal capabilities and is associated with liver protection, antitumor activity, anti-inflammatory activity, and blood sugar and lipid balance [1–7]. As the main active ingredient of *G*. *pentaphyllum*, gypenosides display a typical dammarane-style structure of tetracyclic triterpenes. In higher plants, triterpene saponins are secondary metabolites of isoprenoid compounds. Compared with *Panax ginseng*, *G*. *pentaphyllum* not only generates almost five times higher production of triterpene saponins but also grows faster. Therefore, *G*. *pentaphyllum* is regarded as an economical alternative for producing ginsenosides in the future [8–10].

Sapogenins and sugars, uronic acid and other organic acids constitute the main components of triterpene saponins. The diversity of triterpene saponins is created by oxidation and glycosylation of cytochrome P450 and UDP-dependent glycosyltransferase, respectively, resulting in the modification of the functional groups on the triterpenoid skeleton [11–13]. To better understand the biosynthesis of triterpene saponins in *G*. *pentaphyllum*, we performed transcriptional sequencing of *G*. *pentaphyllum*, which indicated a positive association between the content of triterpene saponins and the expression of key genes (farnesyl pyrophosphate synthase (FPS), squalene synthase (SS), and squalene-2,3-epoxidase (SE)) [14]. However, the expression pattern of key proteins involved in the biosynthesis of gypenosides is still unclear. Therefore, we used differential proteomics to determine the protein sets involved in the synthesis of triterpene saponins and analyzed the expression pattern of important proteins in different tissues of *G*. *pentaphyllum* (roots, stems, and leaves). Understanding the expression patterns of key proteins involved in the biosynthesis of gypenosides in *G*. *pentaphyllum* and their distribution characteristics in different tissues will reveal the mechanism accounting for the diversity of gypenosides.

In recent years, investigations of how modulatory enzymes contribute to the enormous diversity of triterpene saponin structures in *G*. *pentaphyllum* have attracted increasing attention. The CYP and UGT families are the key players in the structural diversity of gypenosides through two reported pathways [15–20]. On the one hand, the cyclic skeleton synthesized by oxidative squalene cyclase (OSC) undergoes site-specific oxidation by CYPs to produce sapogenins with various structures, such as hydroxylation, carboxylation, and even esterification, to form the non-sugar part of saponins. On the other hand, an increased diversity of triterpenoids occurs through the scaffold modification of triterpenoids via UGT-mediated glycosylation. This piqued our interest in further exploring CYPs and UGTs from the transcriptome database and protein quantitative sequencing database of *G*. *pentaphyllum*, which might be involved in the biosynthesis of triterpenoid saponins. RNA-Seq technology provides deep coverage and information on the representation of transcripts, which is very important in non-model plants whose genomes have not been sequenced [11,21]. Hybrid sequencing of the *G*. *pentaphyllum* transcriptome improved the accuracy of prediction of sequences, deducing GpOSC1, GpCYP89, and GpUGT35 were the leading candidates of gypenoside biosynthesis by analyzing the co-expressed genes [11]. However, protein expression level can better reflect the functional state of gene transcripts, prompting us to employ a high-resolution iTRAQ quantitative technique to measure the protein expression of *G*. *pentaphyllum*. Comprehensive analysis of the transcriptome and proteome is effective for finding and cloning some CYPs and UGTs expressed at both the transcriptional and protein levels. Then, qRT-PCR assays were performed to confirm the expression of these candidate genes at the transcription level in different tissues of *G*. *pentaphyllum*. The prediction of the structural characteristics of these CYPs and UGTs and the verification of their tissue expression specificity at the transcriptional level will facilitate future explorations of the regulatory mechanism of the related gene expression.

## Materials and methods

### Protein extraction and digestion

After being ground into powder with liquid nitrogen, powder from *G*. *pentaphyllum* was transferred to a centrifuge tube with lysis buffer (8 M urea, 1% Triton-100, 65 mM dithiothreitol (DTT) and 0.1% protease inhibitor cocktail) and then sonicated three times on ice using a high-intensity ultrasonic processor. Afterwards, the lysis buffer containing the proteins was treated with precooled 15% trichloroacetic acid (TCA) at -20°C for 2 hours and centrifuged to obtain protein precipitates. The protein precipitate was washed with precooled acetone at 4°C for 10 min three times to remove the nonprotein constituents. Next, the purified protein precipitate was redissolved in dilution buffer (8 M urea, 100 mM triethylammonium bicarbonate (TEAB), pH 8.0) for long-term storage. For digestion, the protein solution was reduced with 10 mM DTT for 1 h at 37°C and alkylated with 20 mM iodoacetamide (IAA) for 45 min at room temperature in the dark. For trypsin digestion, the protein sample was diluted by adding 100 mM TEAB to a urea concentration less than 2 M. Finally, trypsin was added at a 1:50 trypsin-to-protein mass ratio for the first digestion overnight and a 1:100 trypsin-to-protein mass ratio for a second 4 h digestion. Approximately 100 μg protein for each sample was digested with trypsin and then frozen for the following experiments.

### iTRAQ labeling and HPLC fractionation

One unit of iTRAQ reagent of protein (approximately 100 μg) was thawed and reconstituted in 24 μl acetonitrile (ACN) by 2-hour incubation at room temperature. The peptide mixtures were pooled and desalted by a StrataX C18 SPE column and then dried by vacuum centrifugation. Afterwards, peptides were separated into 80 fractions with a gradient of 2% to 60% acetonitrile in 10 mM ammonium bicarbonate pH 10 over 80 min. Finally, the peptides were combined into 18 fractions with the aid of an Agilent 300 Extend C18 column and redried by vacuum centrifugation.

### LC-MS/MS analysis

Dried peptides were redissolved in 0.1% formic acid (FA) and directly loaded onto a reversed-phase precolumn (Acclaim PepMap 100) for the separation of peptides. The gradient of the mobile phase was comprised of a 6% to 20% increase in solution B (0.1% FA in 98% ACN) over 22 min, 20% to 35% in 6 min and rising to 80% in 3 min then was held at 80% for the last 4 min, all at a constant flow rate of 300 nl/min on an EASY-nLC 1000 UPLC system. The eluted peptides were treated with nanoelectrospray ionization before analysis by LC-MS/MS (Thermo Fisher Scientific) coupled with the HPLC system.

### Proteomic data processing

LC-MS/MS data were processed by the Mascot search engine (v.2.3.0, https://www.matrixscience.com/) to identify the proteins. Tandem mass spectra were performed for protein identification according to the unigenes of *G*. *pentaphyllum* (67,068 sequences) [14]. Trypsin/P was specified as a cleavage enzyme allowing less than 2 missing cleavages. Mass error was set to 10 ppm for precursor ions and 0.02 Da for fragment ions. Carbamidomethyl on Cys, TMT-6plex (N-term) and TMT-6plex (K) were specified as fixed modifications, while oxidation on Met was specified as a variable modification. The false discovery rate (FDR) was adjusted to ≤ 1%, and the peptide ion score was set ≥ 20. The mass spectrometry proteomics data have been deposited to the ProteomeXchange Consortium (http://proteomecentral.proteomexchange.org) via the iProX partner repository (accession ID: PXD029640) [22].

### Protein annotation and expression analysis

The corresponding protein database was selected for qualitative and quantitative analysis of the above proteomic data from *G*. *pentaphyllum*. Afterwards, repeatability and differential protein function analysis, gene ontology (GO) and metabolic pathway annotation were performed. The differentially expressed proteins in the roots, stems and leaves of *G*. *pentaphyllum* were identified through the following strict screening criteria: proteins with quantitative ratios of expression exceeding 1.5 were deemed to be the upregulated group, while quantitative ratios less than 1/1.5 (0.67) were classified as the downregulated group (p value<0.05). The proteomic data were annotated by the UniProt-GOA database (http://www.ebi.ac.uk/GOA/), and the protein domain was identified by sequence alignment via the InterProScan database (http//www.ebi.ac.uk/interpro/). Next, the InterPro domain database (http//www.ebi.ac.uk/interpro/) was utilized to integrate the predicted protein domain for the subsequent analysis. The online service tool KAAS of the KEGG database (https://www.kegg.jp/) was used for annotating the spliced proteins, while another tool, KEGG mapper, was applied for mapping the annotation results into the corresponding KEGG pathways. Fisher’s precise test was introduced in significance analysis, and the significance level of protein enrichment of each pathway was calculated to determine the pathway with differentially expressed proteins.

### Candidate proteins involved in triterpene saponin biosynthesis

Based on known biosynthesis pathways of triterpene saponins (KEGG pathway ko00900 and ko00909), the candidate proteins could be classified as acetyl-CoA acetyltransferase (AACT), diphosphomevalonate decarboxylase (MVD), 1-deoxyxylulose-5-phosphate synthase (DXPS), isopentenyl diphosphate isomerase (IDI), FPS, geranylgeranyl pyrophosphate synthase (GGPPS), SS, oxidosqualene cyclase (OSC), CYPs and UGTs. According to their protein names and synonyms, they were searched in the annotated results of unique proteins for expression analysis.

### Comprehensive analysis of the *G*. *pentaphyllum* transcriptome and proteome data

To achieve highly consistent comprehensive analyses of the *G*. *pentaphyllum* transcriptome and proteome data, *G*. *pentaphyllum* was collected and packed in the same batch for transcriptome and proteome sequencing. The gene expression analysis results from previous transcriptomics sequencing [14] and the protein expression analysis results from proteomics sequencing were combined to predict the differential expression of genes between the roots, stems, and leaves of *G*. *pentaphyllum*. Enzymes with similar expression trends at both the transcriptomic and proteomic levels were considered to be highly reliable. In addition, GO classification and KEGG pathway annotation of the selected genes (proteins) with significant differences at both the transcriptome level and proteome level were analyzed with R statistics software. Pearson’s correlation analysis was used to evaluate the correlation of the expression at the transcript level (log2 FPKM ratio) and protein levels (log2 iTRAQ ratio) among roots, stems, and leaves in *G*. *pentaphyllum*.

### Identification, cloning and sequence analysis of CYPs and UGTs

We combined NCBI NR annotations of proteome quantitative sequencing results and Swiss-Prot annotations of previous transcriptome sequencing results [14] for screening and identifying enzyme genes that may contribute to the synthesis of triterpene saponins of *G*. *pentaphyllum*. Although many nucleotide sequences were annotated as candidate CYP and UGT genes after screening, most of them were partial cDNA sequences with short lengths. The cDNA lengths of CYP and UGT sequences with complete open reading frames (ORFs) exceeded 1200 bp in the nucleotide database of NCBI (https://www.ncbi.nlm.nih.gov/nuccore). Therefore, we further filtered the annotation results of CYP and UGT genes with an additional standard sequence length (>1200 bp). Finally, confirmed CYP and UGT genes with differential expression at the transcription level (ratio> 2 or <0.5 and P<0.05) were selected for cloning from *G*. *pentaphyllum*.

Fresh *G*. *pentaphyllum* was used to isolate RNA (Takara RNAiso Plus kit) and then reverse-transcribed into cDNA (PrimeScript™ RT reagent Kit with gDNA Eraser, Takara) for gene cloning and quantitative analysis. Primers used for cloning CYPs and UGTs of *G*. *pentaphyllum* are listed in supplementary **S1 Table** (synthesized by GeneCreate). After amplification by PCR and purification by gel electrophoresis, full-length cDNA fragments of CYPs or UGTs were subcloned into the Trans1-T1 plasmid (Transgen) for sequencing (GeneCreate). The sequencing results of CYP and UGT genes were assembled manually and then uploaded to the GenBank database (https://www.ncbi.nlm.nih.gov/genbank/). Nucleotide sequences of CYP and UGT genes were translated into amino acid sequences for homology comparison and motif enrichment analysis by MEGA6 and MEME (http://meme-suite.org/db/motifs), respectively.

### Quantitative real-time PCR analysis

Total RNA of *G*. *pentaphyllum* was reverse transcribed (Promega GoScript™ Reverse Transcription Mix kit with Random Primers) into cDNA for absolute quantitative RT-PCR (SYBR premix Ex Taq™ kit, Takara) analyses with gene-specific primers designed by Primer-BLAST (**S2 Table**). Full-length PCR products of the ORFs of CYP and UGT genes served as standard samples for absolute quantitative RT-PCR assays. Total RNA was used as a template control group to eliminate contamination from the genome. The qRT-PCR program was 95°C for 2 min, 40 cycles of 95°C for 15 s and 60°C for 1 min; a melting curve was then constructed.

## Results

### Proteome analysis of *G*. *pentaphyllum*

**Fig 1:**
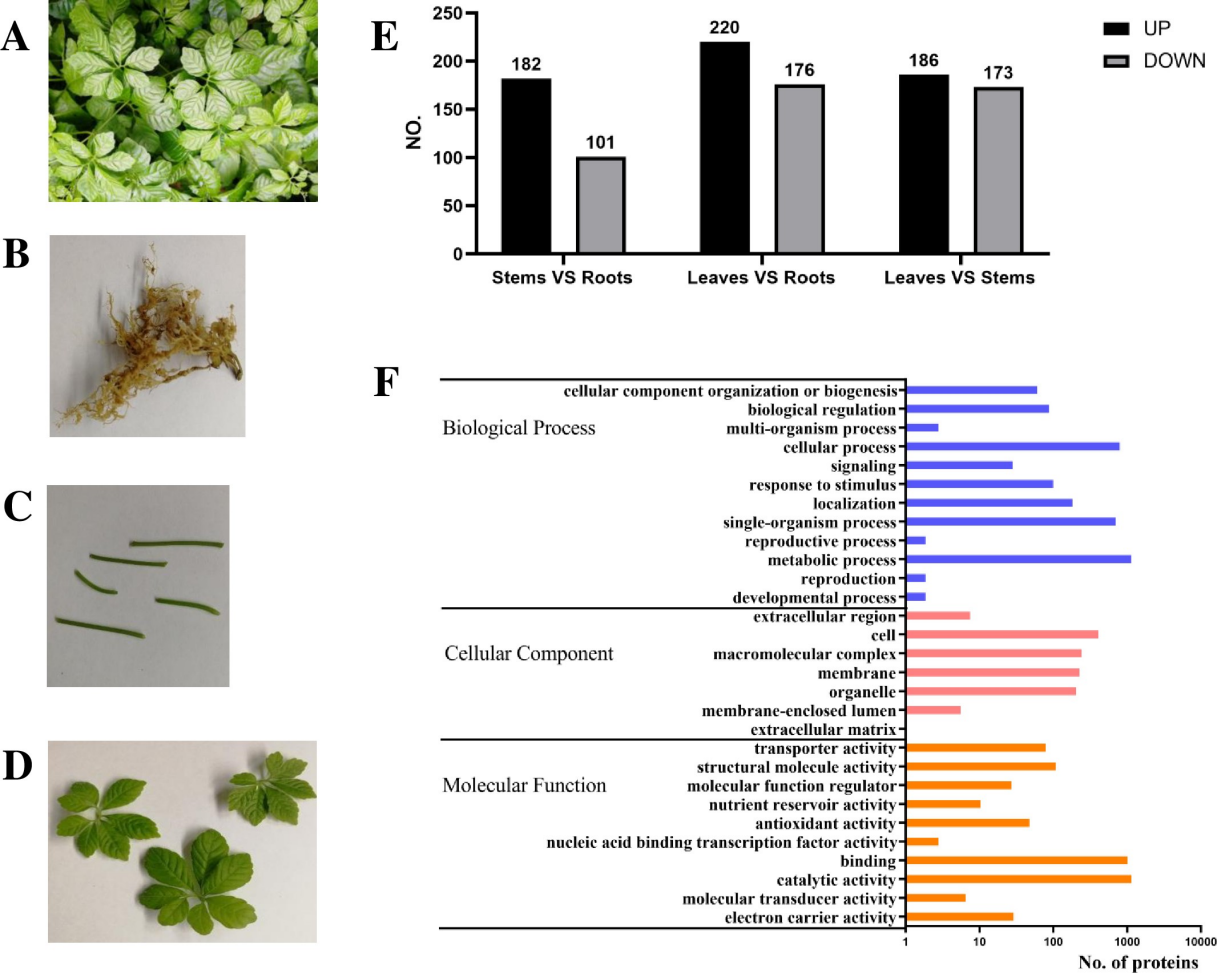
Overview of proteome analysis of *G*. *pentaphyllum*. **(A)** *G*. *pentaphyllum* grown in the medicinal plant garden, **(B)** Roots, **(C)** Stems, **(D)** Leaves, **(E)** Summary of differentially expressed proteins; UP: Upregulated, DOWN: Downregulated. **(F)** GO enrichment analysis of differentially expressed proteins. The enriched GO annotation “biological process,” “cellular components,” and “molecular function” categories are shown. Further classification under each category is also listed. (A-D) Picture by our group. We own the copyright of these pictures, and we give permission to Plos Journal to publish these photos.

*G*. *pentaphyllum* was collected from the Medicinal Plant Garden of Guangxi Traditional Chinese Medical University (Nanning City, Guangxi Autonomous Region of China) in the same batch of transcriptome sequencing. After collection from the natural environment and identification by Mr. Yilin Zhu, the roots, stems, and leaves of *G*. *pentaphyllum* were rapidly separated in two bioreplicates and then frozen in liquid nitrogen for later protein extraction (**Fig 1A–1D**). In total, 3,925 proteins were identified from *G*. *pentaphyllum*, and 2,537 of them were quantified. In this study, a quantitative ratio over 1.5 was considered to be an upregulation, while a quantitative ratio less than 1/1.5 (0.67) was considered to be a downregulation (T test p-value <0.05) **(Fig 1E)**. The differentially expressed proteins among roots, stems and leaves were divided into 29 functional groups, which were divided into three categories: 7 were in the cellular component, 10 were in the molecular function and 12 were in biological process (**Fig 1F)**. These proteins were involved in the functions of catalytic activity, binding, metabolic process, cellular process, and single-organism process, which was consistent with previous transcriptome analysis [14]. After annotation by the KEGG database to identify the most active biological pathways, the differentially expressed proteins were mainly enriched in ribosome, phenylpropanoid biosynthesis, photosynthesis, carbon metabolism, pentose phosphate pathway, glyoxylate and dicarboxylate metabolism, carbon fixation in photosynthetic organisms, cysteine and methionine metabolism and biosynthesis of amino acids.

### Identification of candidate CYPs and UGTs related to triterpene saponin biosynthesis in *G*. *pentaphyllum* by comprehensive transcriptome sequencing and proteome sequencing

**Fig 2:**
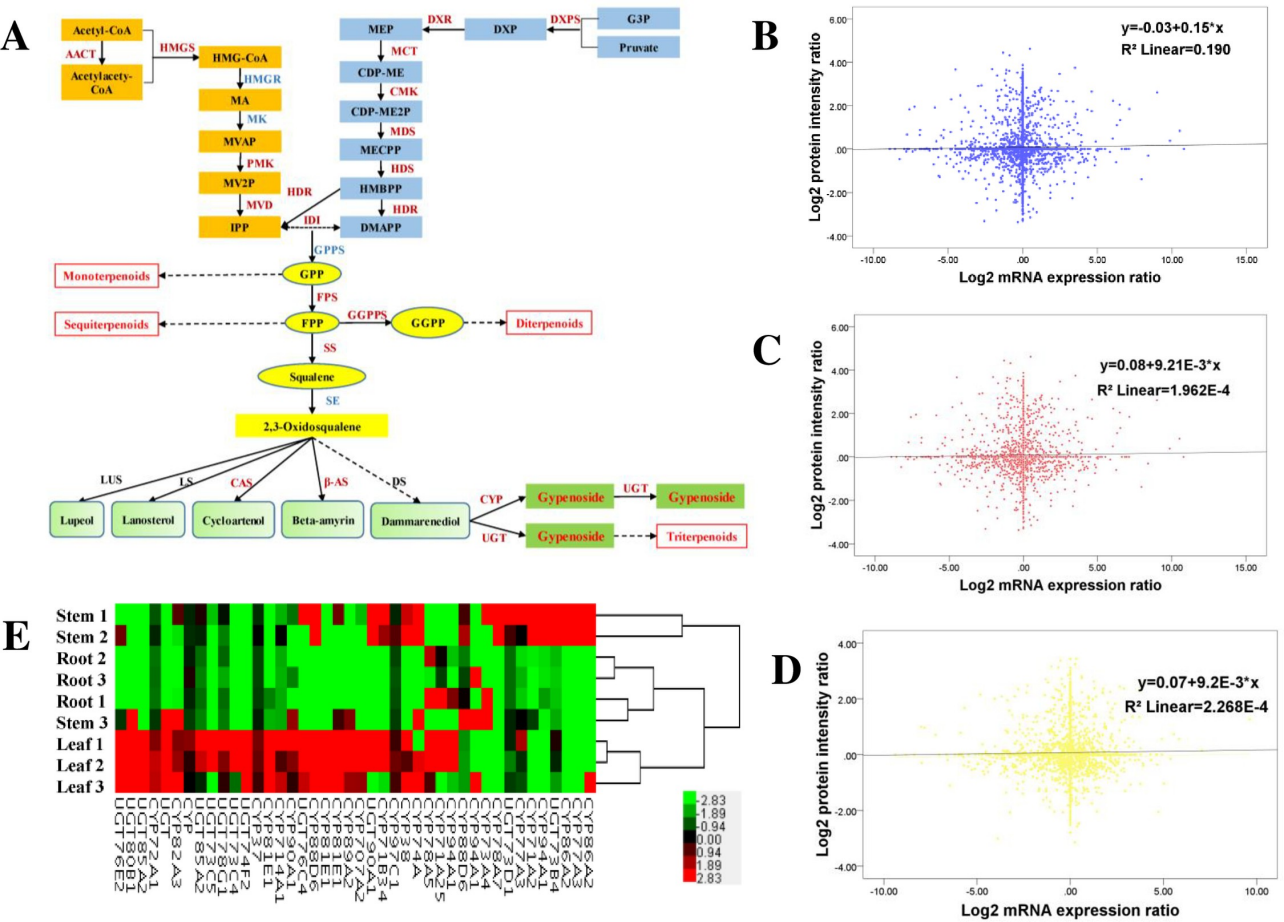
Quantitative proteome sequencing combined with transcriptome sequencing was used to identify genes or proteins in the *G*. *pentaphyllum* triterpene saponin pathway [14]. (A) Triterpene saponin synthesis pathway: Dark red font indicates that the candidate genes or proteins involved in triterpene saponin biosynthesis are identified by transcriptome sequencing and proteomics sequencing at the same time. Blue font indicates the candidate genes involved in triterpene saponin biosynthesis that can only be identified by transcriptome sequencing. LUS: Lupeol Synthase. LS: Lanosterol Synthase. DS: Dammarenediol Synthase. The other gene abbreviations are listed in **S3 Table**. (B-D) Correlation analysis of the overall proteome and transcriptome of *G*. *pentaphyllum*.: (B) Correlation analysis of the overall proteome and transcriptome of Stems vs Roots (Pearson:0.435, p<0.01); (C) Correlation analysis of the overall proteome and transcriptome of Leaves vs Roots (Pearson: 0.014, p = 0.380); (D) Correlation analysis of the overall proteome and transcriptome of Leaves vs Stems (Pearson: 0.015, p = 0.346); (E) Heat Map of Differential Expression of CYPs and UGTs Genes in *G*. *pentaphyllum*: Red represents highly expressed genes. Green represents low expressed genes. Black represents genes with intermediate expression.

To better annotate the triterpene saponin biosynthetic pathway of *G*. *pentaphyllum*, KEGG pathway annotation tool was used to scan the unigenes involved in the target pathway. At the translation level, 36 unigenes were annotated as members of the triterpene saponin biosynthetic pathway from the proteome sequencing results. Almost all published enzymes of triterpene saponin biosynthesis were identified and annotated in the proteome-seq results of *G*. *pentaphyllum* (**S3 Table**). After comparatively analyzing the proteome and transcriptome data, we focused on the 35 unigenes related to triterpene saponin biosynthesis, which were detected in both the transcriptome sequencing results and proteome sequencing results (**Fig 2A**). However, not all unigenes showed consistency in expression analysis at the protein and transcription levels. For instance, c70322_g1_i1 (DXR), c127477_g1_i1 (MCT), and c87813_g1_i1 (GGPPS) were upregulated in leaves at the protein level, but they were not found at the transcriptional level. The fold change at the protein and transcription level among roots, stems, leaves (c127601_g2_i1 (MVD), c70322_g1_i1 (DXR), c127477_g1_i1 (MCT), c123426_g1_i1 (MDS), c102126_g1_i1 (HDR), c122164_g1_i1 (HDR), c136108_g1_i1 (SS), c70785_g1_i1 (β-AS), c132879_g5_i3 (GUT74F2)) was non-proportional (**S4 Table**), which was indicated by the correlation coefficient of the overall proteome and transcriptome data. Pearson’s correlation analysis of Leaves vs Roots and Leaves vs Stems showed weak or no correlation, indicating that the difference at the transcription level is basically independent of the difference at the corresponding protein level. The transcriptome sequencing results and the quantitative protein sequencing results of Stems vs Roots were moderately correlated with high consistency (**Fig 2B–2D**). This finding also explains the necessity of sequencing the transcriptome and proteome of *G*. *pentaphyllum*. This moderate correlation may be because protein expression levels are regulated by a variety of posttranscriptional, translational and posttranslational mechanisms. The other reason for this effect may be due to a feedback loop between mRNA translation and protein degradation. By comparing the genes detected in the transcriptome with the proteomic data, we found that high-abundance transcripts could be easily detected at the protein level, while most of the low-abundance transcripts were not detected in the proteome data. In fact, **Fig 2A** shows that most of the candidate genes of enzymes (proteins) involved in triterpene saponin synthesis can be annotated both in transcriptome and proteome sequencing data, such as AACT, HMGS, PMK, MVD, FPS, SS, CAS, and β-AS. Only a few candidate genes were annotated in the transcriptome data but not in the proteome, such as HMGR, GPPS, and SE. Further analysis is needed for the inconsistency of the expression trends of some candidate genes related to triterpene synthesis in the transcriptome and proteome of different parts of *G*. *pentaphyllum* roots, stems and leaves.

The diversity of CYP and UGT supergene families accounted for the structural and functional diversity of gypenosides. Based on the previous *G*. *pentaphyllum* transcriptome data obtained by our research group, 641 CYP records and 178 UGT records were first screened through Swiss-Prot annotations, while 95 CYP records and 30 UGT records with qualified sequence lengths (ORFs>1200 bp) were treated as candidate CYP and UGT genes. Then, according to the NR annotation of proteome sequencing, we accumulated 89 CYP sequences and 31 UGT sequences (tested and verified through Protein-BLAST and duplicate sequences were removed). By counting the RPKM values of CYP- and UGT (ORF>1200 bp)-expressing differential genes, overall, the expression of *G*. *pentaphyllum* CYP and UGT genes was relatively higher in leaves and stems and lower in roots (**Fig 2E**). Compared with roots, stems showed a closer expression pattern of CYP and UGT unigenes, similar to leaves. We obtained 17 CYP unigenes and 11 UGT unigenes with differential expression by transcriptome sequencing and tried to clone them from *G*. *pentaphyllum*. Finally, 7 CYP and 5 UGT were chosen for further qRT-PCR analysis.

### Cloning and sequence analysis of *G*. *pentaphyllum* CYPs and UGTs

**Fig 3:**
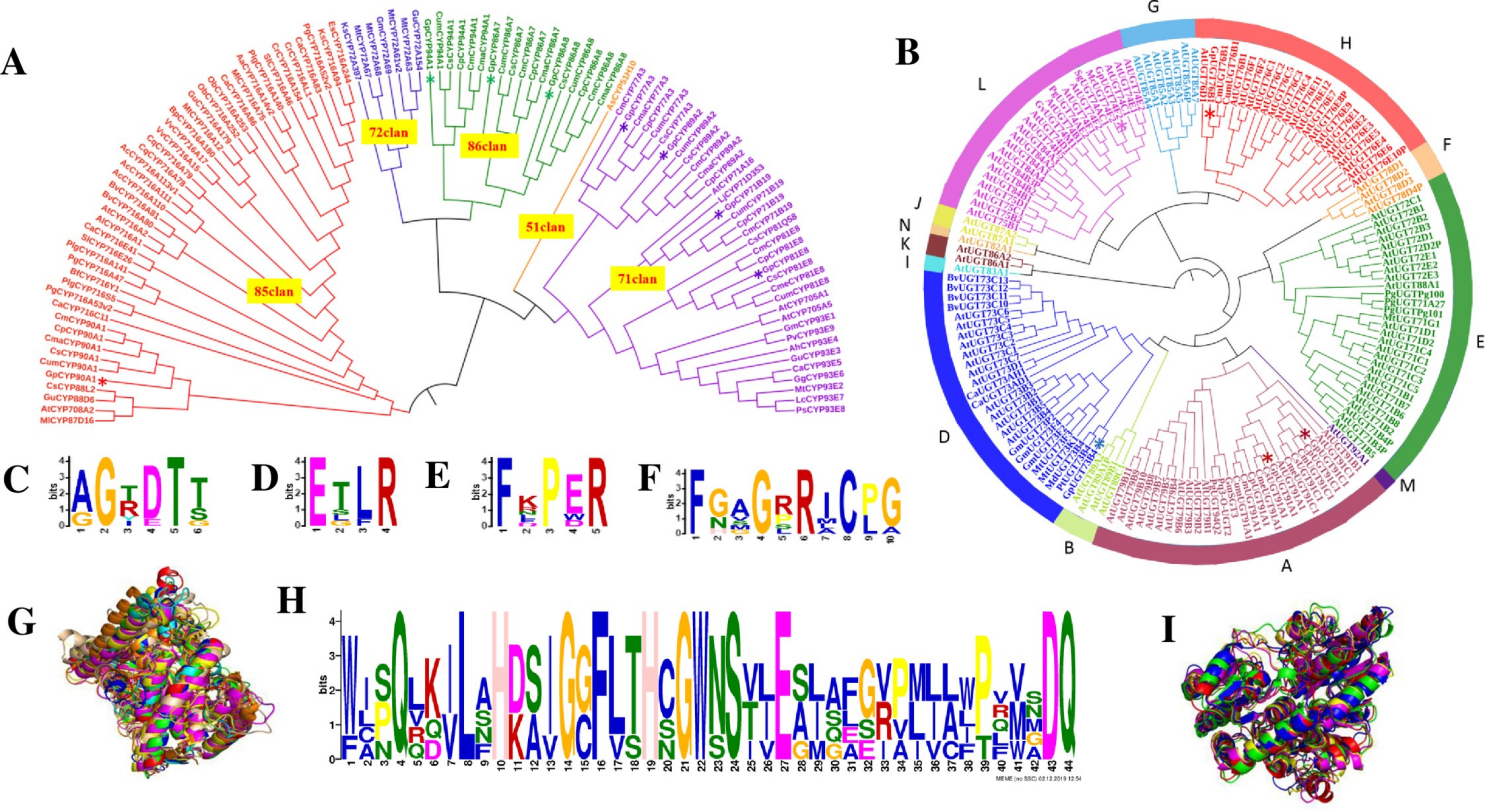
Cloning and sequence analysis of *G*. *pentaphyllum* CYP and UGT genes. (A) CYP phylogenetic tree analysis of *G*. *pentaphyllum*, Cucurbitaceae and other plants, the functional activities of which were identified [17]. *denotes the CYP protein of *G*. *pentaphyllum*. Gp: *Gynostemma pentaphyllum*. At: *Arabidopsis thaliana*. Pv: *Phaseolus vulgaris*. Ps: *Pisum sativum*. Lc: *Lens culinaris*. Gg: *Glycyrrhiza glabra*. Ca: *Cicer arietinum*. Ah: *Arachis hypogaea*. Gu: *Glycyrrhiza uralensis*. Mt: *Medicago truncatula*. Gm: *Glycine max*. Ml: *Maesa lanceolate*. Ks: *Kalopanax septemlobus*. Lj: *Lotus japonicas*. As: *Avena strigose*. Al: *Arabidopsis lyrata subsp*. Lyrata. *Aa*: *Artemisia annua*. Vv: *Vitis vinifera*. Sl: *Solanum lycopersicum*. Pg: *Panax ginseng*. Cq: *Chenopodium quinoa*. Bv: *Barbarea vulgaris subsp*. *Arcuate*. Ca: *Centella asiatica*. Ac: *Aquilegia coerulea*. Plg: *Platycodon grandifloras*. Cr: *Catharanthus roseus*. Bp: *Betula platyphylla*. Es: *Eleutherococcus senticosus*. Ob: *Ocimum basilicum*. Bf: *Bupleurum falcatum*. Cs: *Cucumis sativus*. Cp: *Cucurbita pepo subsp*. *Pepo*. Cm: *Cucurbita moschata*. Cum: *Cucumis melo*. Cma: *Cucurbita maxima*. Cme: *Cucumis melo var*. *makuwa*. (B) UGT phylogenetic tree analysis of *G*. *pentaphyllum*, Arabidopsis thaliana, Cucurbitaceae and other plants, the functions of which were identified [26]. *denotes the UGT protein of *G*. *pentaphyllum*. (C) Results of the MEME repeat of the corresponding CYP protein AGxDTT motif of *G*. *pentaphyllum* and *Cucurbitaceae*. (D) Results of the MEME repeat of the corresponding CYP protein ExxR motif of *G*. *pentaphyllum* and *Cucurbitaceae*. (E) Results of the MEME repeat of the corresponding CYP protein PER motif of *G*. *pentaphyllum* and Cucurbitaceae. (F) Results of the MEME repeat of the corresponding CYP protein FxxGxRxCxG motif of *G*. *pentaphyllum* and *Cucurbitaceae*. (G) Prediction of CYP protein tertiary structure of *G*. *pentaphyllum*: Red: CYP71B19; Green: CYP77A3; Yellow: CYP86A7; Magenta: CYP86A8; Cyan CYP89A2; Orange: CYP90A1; Light pink: CYP94A1. (H) Results of the MEME repeat of the corresponding UGT protein PSPG motif of *G*. *pentaphyllum* and *Cucurbitaceae*. (I) Prediction of the UGT protein tertiary structure of *G*. *pentaphyllum*: Red: UGT73B4; Green: UGT74F2; Blue: UGT76B1; Yellow: UGT91A1; Pink: UGT91C1.

CYP and UGT genes were amplified by PCR and purified by agarose gel electrophoresis. After sequencing and assembly, 7 CYP cDNAs and 5 UGT cDNAs with complete ORFs were uploaded to GenBank. The ORF length of these cDNAs ranged from 1365 bp to 1644 bp, and the genes and accession numbers are shown in **S5 Table**. We used Editseq software to translate the complete ORF sequences of CYP and UGT into amino acid sequences for further analysis. These ORFs encoded polypeptide chains with lengths of 455~548 amino acids. The phylogenetic tree of CYP was constructed by the CYP amino acid sequences of *G*. *pentaphyllum*, Cucurbitaceae and other plants with verified CYP amino acid sequences. The phylogenetic tree showed that the CYP amino acid sequences of *G*. *pentaphyllum* were divided into groups 71, 85, 86 and that *G*. *pentaphyllum* had a closer relationship with *Cucurbita moschata*, *Cucurbita pepo subsp*. *Pepo*, *Cucumis sativus*, *Cucumis melo* (**Fig 3A**). Our previous studies also showed that the FPS, SS, and SE of *G*. *pentaphyllum* had closer genetic relationships with the corresponding genes of Cucurbitaceae plants [14,23,24]. We constructed the UGT phylogenetic tree among *G*. *pentaphyllum*, *Cucurbitaceae*, *Arabidopsis thaliana* and other species with verified UGT amino acid sequences. Some of them were divided into groups A, D, E, F, G, H, and L, which were suggested to participate in the glycosylation of plant triterpene saponins [25]. UGT unigenes of *G*. *pentaphyllum* were divided into groups A, D, H, and L [26] (**Fig 3B**).

We used DNAMAN software to calculate and display the homology of CYP and UGT (**S1 and S2 Figs**). CYP is a large superfamily of heme-containing monooxygenases involved in the oxidative metabolism of multiple substrates. Despite the low similarity of sequence identity, CYP members still have a conserved structural core [27]. The MEME repeat of the CYP protein motifs of *G*. *pentaphyllum* and Cucurbitaceae showed that the peptide chain contained the conserved motifs AGxDTT, ExxR, PER and CxG (**Fig 3C–3F**). AGxDTT, also known as the oxygen binding domain (OBD), contributes to the binding and activation of oxygen [28,29]. ExxR and PER patterns form an E-R-R triplet, which is very important for locking the structure of the heme pocket in place and then ensuring the stability of the core structure [30]. The sequences of CYPs contained a highly conserved peptide (FxxGxRxCXG), which is the typical and unique thiolate ligand of all cytochrome P-450 heme [31]. Located in the middle position of CxG, the inside amino acid was regarded as the decider for the structure, activity and substrate specificity of P450 [32]. Although the CYP sequences of *G*. *pentaphyllum* did not show high similarity, the 3D structures of these CYPs were highly consistent (**Fig 3G**). As shown by the alignment of *G*. *pentaphyllum* UGTs, the glycosyltransferase PSPG motif consisted of 44 conserved amino acid sequences at the C-terminus, which may bind uracil nucleoside-5’-diphosphate-glucose (UDPG) during glycosylation (**Fig 3H**) [33]. Osmani SA proved that 22 (W), 43 (E/D) and 44 (Q) residues of GT1 of *Vitis vinifera*, UGT71G1 and UGT85H2 of *Medicago trunkatula*, and UGT72B1 of *Arabidopsis thaliana* were involved in the formation of hydrogen bonds with glucose groups, which were also found in *G*. *pentaphyllum* UGTs [34]. Although the similarity of UGT sequences of *G*. *pentaphyllum* is not high, a high consistency in 3D structure was observed from the comparison between UGT sequences of *G*. *pentaphyllum* and other known UGTs (**Fig 3I**).

### Differential expression of CYPs and UGTs in different tissues of *G*. *pentaphyllum*

**Fig 4:**
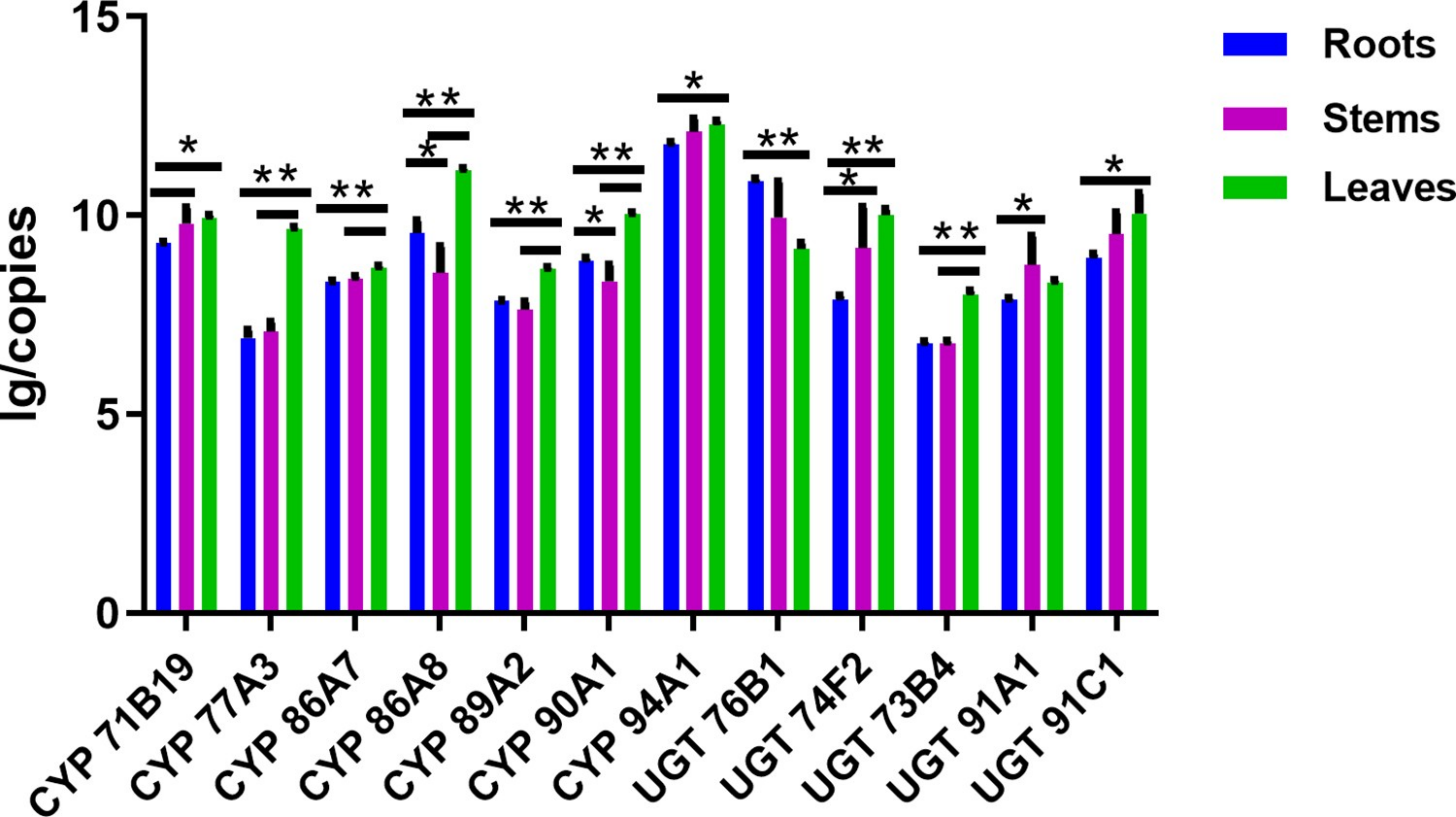
Comparison of qPCR copy values of CYPs and UGTs of *G*. *pentaphyllum* roots, stems and leaves. * means p<0.5; ** means P<0.01.

qRT-PCR analysis of CYPs and UGTs showed that the transcriptional expression of CYP and UGT genes among the roots, stems and leaves of *G*. *pentaphyllum* was tissue-specific (**Fig 4**). Some CYPs and UGTs had higher transcriptional expression in leaves than in stems or roots. The distribution tendency of CYPs and UGTs in various tissues showed the following: leaf>stem>root, such as CYP77A3, CYP86A7, CYP94A1, UGT74F2, and UGT91C1; leaf>root>stem, such as CYP86A8, CYP89A2, CYP90A, and UGT73B4. In the latter case, the expression of most post-modification enzyme genes was not significantly different between roots and stems, while individual genes, such as UGT91A1 and UGT76B1, even had higher expression in stems and even roots. Since CYPs and UGTs are very large gene superfamilies in plants, generally, not all their expression patterns are absolutely consistent with other key enzyme genes, such as FPS or SS, in triterpene saponin biosynthesis studies (leaves > stems > roots) [14].

## Discussion

*G*. *pentaphyllum* is well known for its abundant and diversiform triterpene saponins compared with other medicinal herbs. We performed transcriptome sequencing in *G*. *pentaphyllum* to understand the key enzymes and vital pathways involved in the biosynthesis of triterpene saponins [14]. Transcriptome sequencing data indicated that three key enzymes involved in the biosynthesis of triterpene saponins (FPS, SS and SE) showed the same expression pattern (leaf > stem > root), which was coincident with the distribution of triterpene saponins [14]. Inspired by the above findings, we attempted to investigate the genes and pathways that accounted for the huge structural diversity of triterpene saponins by the multi-analysis of transcriptome and proteome sequencing data. Because of the decisive contribution of CYP and UGT superfamilies in the formation of triterpene saponins with various structures, 7 CYP genes and 5 UGT genes of *G*. *pentaphyllum* with high-quality sequences were finally identified, cloned and functionally annotated after performing multi-analysis of the transcriptome and proteome sequencing data. This was the first report exploring CYPs and UGTs of *G*. *pentaphyllum* with identified sequences. Except for UGT76B1 and UGT91A1, other CYPs and UGTs showed similar expression trends among leaves, stems and roots, as verified by qRT-PCR assays (**Fig 4**). Finally, we performed bioinformatics analysis on the sequences of CYPs and UGTs to further understand their vital roles in the structural diversity of triterpene saponins.

We also found that the correlation between the proteome and transcriptome sequencing was not as tight as expected. In theory, there should be a strong correlation between the proteome and transcriptome of tissues with the same growth conditions and growth environments. High levels of mRNA generally lead to a high abundance of protein and vice versa. However, many published studies have reported the relatively low consistency of expression patterns between transcriptome and proteome sequencing [35–38]. There are two possible reasons to account for this discrepancy: one is the various forms of protein expression regulation (posttranscriptional, translational and posttranslational), and the other is a feedback loop between mRNA translation and protein degradation. In addition, the difference in annotation abundance between the transcriptome and proteome may be due to technical reasons, resulting in a weak correlation between them. Transcripts with high abundance are readily detected in the proteome; otherwise, they are difficult to detect.

The structures of triterpene saponins are highly obscure due to the oxidation and glycosylation performed by post-modification genes for enzymes. Post-modification enzymes not only have low concentrations in plants but also show complex features with similar structures. It is difficult to obtain a large amount of triterpene saponins from medicinal plants through separation, extraction or chemical synthesis, which further limits the wide application of triterpene saponins [39]. Currently, synthetic biology provides new ideas for the large-scale production of high-value natural products. Understanding the biosynthetic pathway of gypenosides with identified key enzyme genes provides the possibility for obtaining valuable triterpene saponins through synthetic biology methods [40]. Therefore, it is logical to identify, clone and functionally analyze the CYPs and UGTs of *G*. *pentaphyllum* to promote biological research and commercial application of triterpene saponins.

Not all post-modification enzyme genes showed the same expression pattern among various tissues in this study. The encoded products of CYPs and UGTs are not only diversified in structure but also differ in substrate specificity. Recognition ability may tend to catalyze the formation of one or several specific monomeric saponins. Although the content of saponins varies in different tissues of *G*. *pentaphyllum*, it does not signify that post-modification enzyme genes for the synthesis of one or several monomeric saponins are differentially expressed at the transcriptional level in different tissues [41,42]. It is not surprising that the differential distribution of triterpene saponins cannot infer the differential expression of candidate genes involved in the biosynthesis of gypenosides. To further explore the correlation between the expression of post-modification enzymes (CYPs, UGTs) and gypenoside synthesis, methyl jasmonate (MeJA) could be used to induce the expression of these key enzyme genes. Meanwhile, it should be investigated whether the relationship is consistent between the changes in the content of gypenosides and the expression of CYPs and UGTs. Therefore, the identification of candidate genes involved in the biosynthesis of gypenosides still needs continuous research efforts.

CYP71B19, CYP86A7, CYP94A1, UGT74F2, UGT91A1, and UGT91C1 were identified from both transcriptome sequencing and proteome sequencing with good sequence quality. We performed qRT-PCR assays to verify gene and protein expression predicted by transcriptome sequencing and proteome sequencing, respectively. The gene expression results from qRT-PCR assays basically met the expression pattern of key genes from transcriptome sequencing results. The protein expression of UGT74F2 showed an expression tendency (root < stem < leaf), which was replicated in transcriptome sequencing. However, the expression of CYP71B19, CYP86A7, CYP94A1, UGT91A1 and UGT91C1 was undetected in the proteome sequencing results due to their low expression levels. Biomass accumulation of the post-modification enzyme protein takes a long time. If the key genes of the triterpene synthesis pathway were transformed into model species such as yeast for engineered expression, it would be expected that specific triterpenes of *G*. *pentaphyllum* would be produced on a larger scale. This study lays the groundwork for further identifying the specific monomers of triterpene saponins that regulate the expression of related genes.

## Supporting information

S1 Fig Amino acid sequence homology alignment results of *G*. *pentaphyllum CYPs*. (PPTX)

S2 Fig Results of amino acid sequence homology alignment of *G*. *pentaphyllum UGTs*. (PPTX)

S1 Table Primers list of CYPs & UGTs amplification (full-length cDNA). (XLSX)

S2 Table qPCR primers list of CYPs and UGTs. (XLSX)

S3 Table List of the detected proteins in triterpene saponins biosynthesis of *G*. *pentaphyllum*. (XLSX)

S4 Table Standardized expression of CYPs and UGTs. (XLSX)

S5 Table List of CYP and UGT accession number. (XLSX)
