# Supplementary material for: Gene excavation and expression analysis of CYP and UGT related to the post modifying stage of gypenoside biosynthesis in Gynostemma pentaphyllum (Thunb.) Makino by comprehensive analysis of RNA and proteome sequencing
Source: PLoS One. 2021 Dec 7;16(12):e0260027. doi: 10.1371/journal.pone.0260027 (PMC8651138; doi:10.1371/journal.pone.0260027)
Supplement: S1 Fig — (PPTX) [file pone.0260027.s001.pptx]

## Slide 1
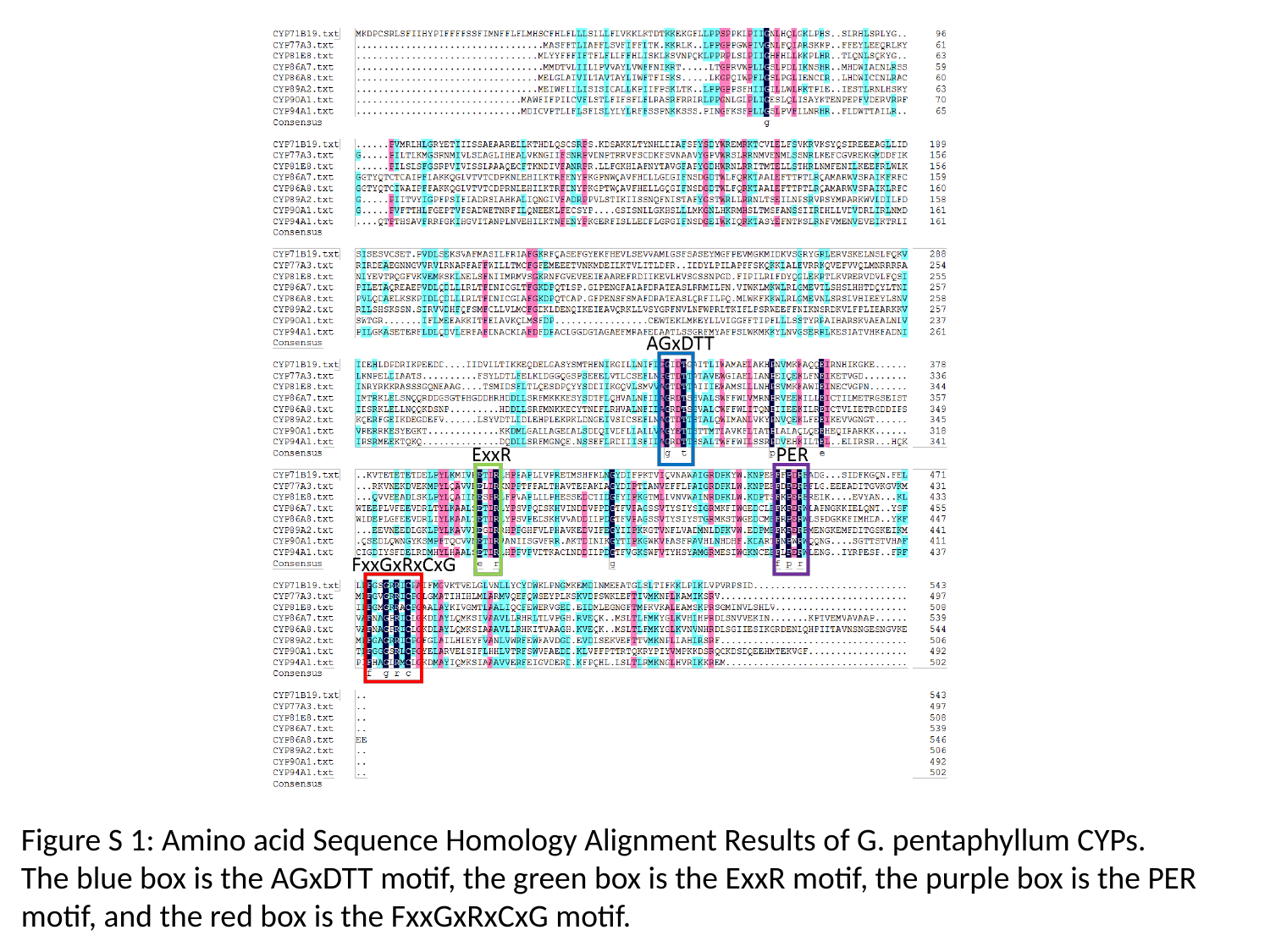

Figure S 1: Amino acid Sequence Homology Alignment Results of G. pentaphyllum CYPs.
The blue box is the AGxDTT motif, the green box is the ExxR motif, the purple box is the PER motif, and the red box is the FxxGxRxCxG motif.
