# Supplementary material for: Gene excavation and expression analysis of CYP and UGT related to the post modifying stage of gypenoside biosynthesis in Gynostemma pentaphyllum (Thunb.) Makino by comprehensive analysis of RNA and proteome sequencing
Source: PLoS One. 2021 Dec 7;16(12):e0260027. doi: 10.1371/journal.pone.0260027 (PMC8651138; doi:10.1371/journal.pone.0260027)
Supplement: S2 Fig — (PPTX) [file pone.0260027.s002.pptx]

## Slide 1
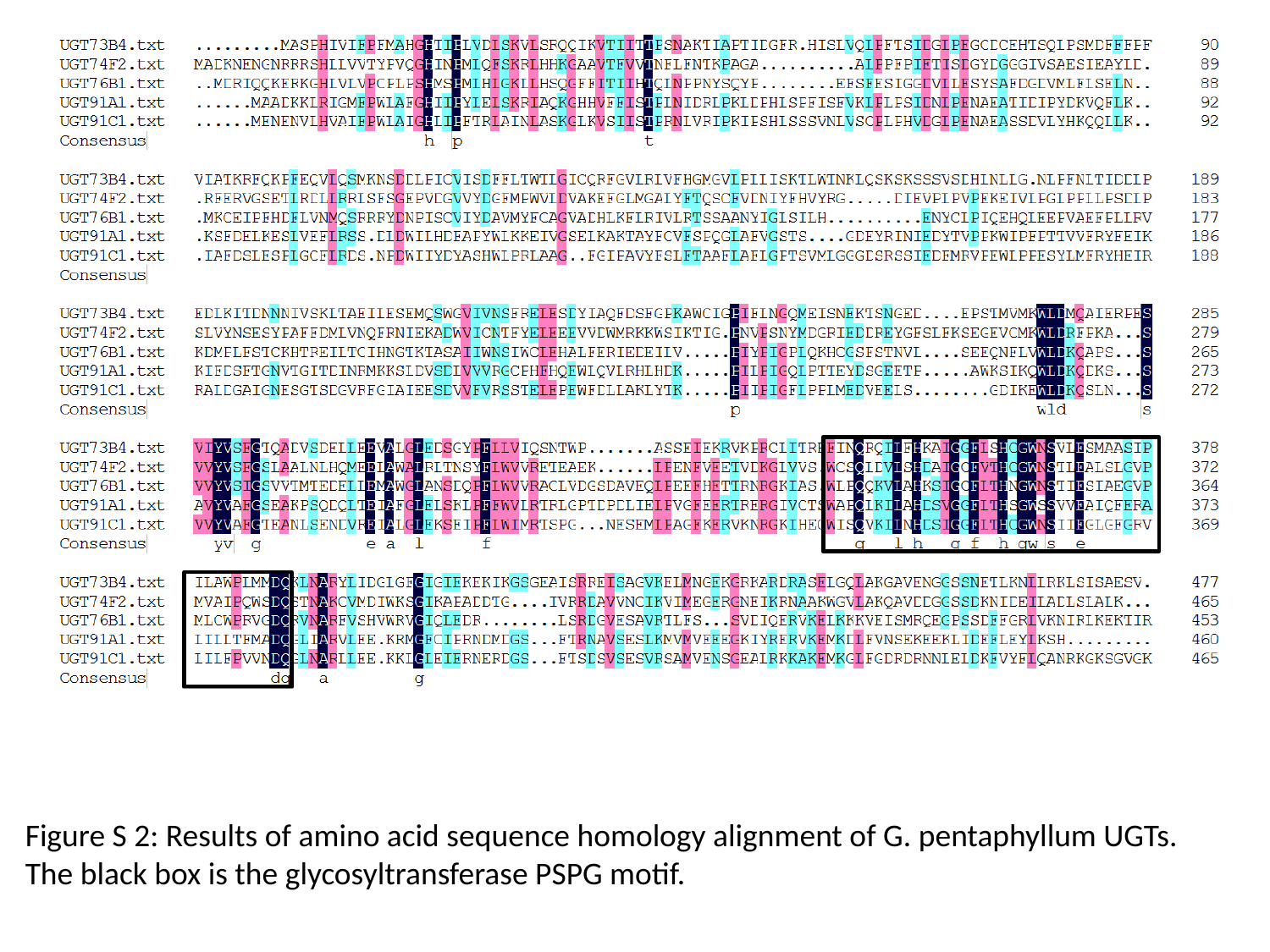

Figure S 2: Results of amino acid sequence homology alignment of G. pentaphyllum UGTs.
The black box is the glycosyltransferase PSPG motif.
